# On the Recent Advances in the Theory of Vision

**Published:** 1872-10

**Authors:** H. Helmholtz, F. W. Abbott

**Affiliations:** The Royal Society of London


### Translations.

### *On the recent Advances in the Theory of Vision. By* H. HELMHOLTZ, *(of the Royal Society of London.)*

Translated from *Les Annales d' Oculistique*, By F. W. ABBOTT, M. D.

Besides the knowledge which is founded on ideas and consequently lends itself to expression in words, our mind is capable of an order of operations in which it works only upon sensual impressions, which cannot be translated into words. The result of these operations is what we call *cognizance* (le connaitre). We cognize, (connaissons) a man, a road, an article of food, an odoriferous substance; which means that we have either tasted or smelled or perceived these objects, that we preserve in the memory this sensual impression so as to be able to recognize it upon occasion, and this we do without our being able to give a description of it in words. It is not less certain that this cognizance is susceptible of as great a degree of precision and accuracy as a knowledge susceptible of being expressed in words. But we have to do here with some thing which we can impart only by presenting the objects themselves, or by imitating the impression which they produce; it is in this manner that a portrait makes known the aspect of a person,

An important part of the cognizance (le connaitre) is the knowledge (connaissance) of the innervation which we must employ to obtain any result whatever, by the motions of our limbs and our organs. Every one knows that it was necessary for him to learn to walk; that he became able afterwards to walk upon stilts, to skate, to ride, to swim, to sing, to learn the sounds of a strange language etc. By observing nurses we perceive very soon that they have to teach a whole series of things which it afterwards appears to us impossible not to have known always, such as to turn the eyes to a light which we wish to see. This kind of cognizance (*connaitre*) is called knowledge (*savoir*). We must not confound know (*savoir*) with can *(pouvoir*) (*kennen and koennen*) as the Germans sometimes do, on account of the analogy which these words present in their language. Let us remark that this knowledge of the effects of volition must attain an extraordinary degree of accuracy and security for it to be possible for us to keep our equilibrium on stilts or in skating, or to attempt with the voice or upon a violin a tone which would become false with a demi-vibration per hundred more or less.

It is clear that our mind may combine the memory of sensual impressions according to the mode which it employs ordinarily to combine words, and thus form some thing analogous to what in spoken language is called a proposition or a judgment. It is in this manner that I may know that a man with whose figure I am acquainted possesses a singular voice, with the tone of which I am well acquainted. I would recognize his figure and his voice without hesitation among a thousand, and the one immediately brings up the other. But it is impossible for me to express this relation in words, if I have not at my disposal other expressible circumstances to define the man in question. Then I can only flank the difficulty and say: The voice which we are hearing now belongs to the man whom we saw such a day, at such a place.

In certain general propositions, as well as in particular propositions, words may be replaced by sensual impressions; let us be content with citing the effects of arts of imitation. The statue of a divinity could not give me the impression of a certain character, temperament, or determinate disposition of mind, if I did not know that the expression or the gesture which it presents possesses this signification in the majority of cases. Not to depart from the domain of sensual perceptions, if I know that a certain way of looking goes with fixing a point two feet distant and to the right, and if I had an accurate knowledge of the degree of innervation necessary for this effect, here is a general proposition which is applicable to all the cases where I have fixed, or rather where I may fix, a point situated in the manner indicated. This proposition which cannot be expressed in words, is the result which comprises what I have learned by the concordance of my anterior experiences. It may become at any moment the *major* of a syllogism, when I happen to fix a point situated as has just been said, and which I perceive that I look at conformably to that major. The *minor* consists in perceiving that I look in this manner, and the *conclusion* consists in saying that the object seen is situated at the corresponding place.

Let us suppose now that I look in this manner, but through a stereoscope. I know that there is not before me any real object at the place in question, but none the less do I experience the same sensual impression as if there were one there: and this is an impression which, neither by words nor for myself, can I define otherwise than by saying that this impression is what I would receive in a normal manner of looking, if a real object were at that place. Let us insist on this point. The Physiologist can without doubt describe what passes here as it relates to the position of the eyes, the position of the retinal images, etc. But it is impossible to characterize and define immediately the sensation otherwise than as has been said above. We know that we have to do with an illusion of the sensation, nevertheless we cannot destroy the sensation of this illusion. This means that it is impossible for us to set aside the memory of the normal significance of a sensation, even when we know that this signification ceases to be applicable: this is no more possible for us than not to think of the signification of a word of our mother tongue, in whatever signification it may be pronounced.

If these reasonings, relating to sensual perceptions, present themselves to us as irresistibly, as any other natural external force, and if their results then seem to be given by an immediate perception, independent of our participation, this is not a reason for considering them as being of another nature than logical, conscious reasonings, or at least than those which truly merit this name. That which we can do voluntarily and wittingly to form a conclusion is limited to bringing together the elements which constitute the reasoning. When these elements are really complete, the conclusion is irresistably forced upon us. The reasonings which we think that we can turn in this way or that at will, in general do not avail much.

We see that our researches lead us into the domain of psychical actions, which has scarcely been occupied as yet as an object of scientific research, because it is difficult to find expressions in which to speak of them. They have been considered rather in esthetic researches where they play a great role under the name of "intuitive verities," "unconscious reasonings" "perceptive intelligence" and similar vague expressions. It is prejudice which considers these psychical acts obscure, vague and *inconscient*, regards them as purely mechanical operations, and assigns them to a class inferior to that of conscious thought which can be expressed in words. I do not think that any difference can be demonstrated between the natures of these actions. The immense superiority of knowledge, perfected up to the point of being able to be expressed, is sufficiently explained in other ways: on the one hand, language furnishes the possibility of uniting and preserving the experiences of millions of individuals and thousands of generations, and of rendering them at the same time more and more certain and more and more general by a continual verification; on the other hand it is upon speech that the power rests which men have of uniting and acting in common, which forms the greatest part of their power. In these two relations cognizance (connaitre) cannot vie with knowledge (savoir), without its following of necessity that it must be less clear or of a different nature.

The partisans of naturalistic theories invoque the aptitudes of new born animals, many of which show themselves to be more skilful than the infant. It is certain that in spite of the more considerable development of the brain and the superiority of his intellectual perfectibility, it takes the child a long time to become capable of the simplest acts, as for example, to direct his eyes towards an object, or to grasp what he sees. Should we not conclude from this that the child has much more to learn than the animal, guided by the instincts in which, so to speak, it is penned ? They say that the calf sees the udder of the cow and goes to find it; it remains to be seen whether it does not merely smell it, and continue the motions which cause it to approach this odor. It is certain that the infant does not see the breast; it often turns obstinately away from it to seek it in the other direction; The little chicken commences at once to pick to find grains; but it has already pecked in the shell, and it seems to pick at hap-hazard at the first, when it sees the mother set the example. When by chance it has met with some grains it may then learn to observe their appearance, and this the more quickly since all that it is necessary for it to learn in life is very limited. New observations on this subject are desirable, for the purpose of elucidating the question under consideration. The observations hitherto made do not appear to me to prove that animals at birth show forth anything except tendencies, and it is very certain that man shows distinctly that these innate tendencies are reduced in him to the smallest possible measure.

Our mind proceeds, moreover, in an entirely analogous manner, when it has to do with another system of signs whose choice is arbitrary, and for the comprehension of which the intervention of education consequently is evident: I mean the words of the mother tongue.

To learn a first language is evidently a much more difficult thing than to learn a foreign language in later years. It is necessary, first of all, to divine that what one hears are signs; at the same time it is necessary to discover the meaning of each word by an induction of the same kind as that which has taught one to interpret sensations. Nevertheless we see children of a year old commencing to comprehend, if not to repeat, certain words and certain phrases. Even dogs are known to attain this.*

This relation between the name and the object, which is evidently a result of education, becomes as indistructible as the relations between the sensations and the objects.

We are not able to escape thinking of the normal signification of a word even when, as an exception, it has been used in another meaning. We cannot suppress the emotion which a romance produces, even when we know that we have to do with a fiction ; it is for the same reason that we cannot efface from our mind the normal signification of the sensations in the case of the most clearly recognized illusion of the senses.

There is, finally, a third point of comparison which merits our attention. The elementary signs of our language are embraced in twenty-six letters and what limit is there to the variety of the ideas which their combinations enable us to express! Let us think now of the immense richness of the elementary signs which the visual nervous apparatus may furnish. We may estimate the number of fibres of the optic nerve at 250,000, each one of them may receive infinitely varied degrees of irritation, proceeding from one or even from three fundamental colors. It is plain that here there is where with to form a system of combinations infinitely more rich than with the few letters of our alphabet, without speaking of the rapid variations which the visual images may undergo. Hence it should not astonish us that the language of our senses gives us information infinitely more particular, more individuated and with finer shades of meaning than spoken language can give.

Such is the solution of the problem, and the only one, it seems to me, which the facts at present known enable us to accept. The circumstances in which we have found the most marked disagreement between the sensations and the object, either qualitatively, or in the relation of the localization, have been the most instructive for us, because these are what has led us into the right way. Even those Physiologists who try to save the debris of that theory which supposes a preestablished harmony between the sensations and the real objects are forced to admit, that sensual perception only attains its highest degree of perfection when it has experience for a foundation: they are even forced to admit that it is this which finally prevails when it comes in conflict with the pretended natural conformity of the organ with the objects. Consequently we can no longer attribute to the conformation of the organ any other role than that of favoring, perhaps, at their origin the formation of our notions.

The harmony between the visual perceptions and the external world rests therefore entirely, or at least essentially, upon the same basis as all the rest of our knowledge of the real world, that is to say, upon experience constantly verified by new experiences, such as are produced by the motions of our body. It is evident that the harmony between the real world and our sensations is demonstrated to us within the boundaries where are enclosed the experiences which our senses furnish us, but this is precisely the whole of what is necessary for us in practice. Beyond these limits, on the question of properties, for example, we can demonstrate that there is disagreement. The relations of time, place, equality, and those of number, size and law, in short all that is mathematical, are common to the external and the internal world, and for all these relations we may look for a perfect concord between the representations and the objects. I think that we will not be angry with nature for having hidden the profundity, so difficult to appreciate, of these abstractions under the infinite variety of the signs by which objects manifest themselves to our senses. If the abstractions escape us, the signs are only the more appreciable and more rapidly utilisable in practice; which will not hinder a speculative spirit from finding still sufficient indications for distinguishing between what is sign and what is image.

